# Prognostic value of the expression and localization of cell proliferation and apoptosis markers in unicystic ameloblastomas

**DOI:** 10.1038/s41598-024-54132-7

**Published:** 2024-02-15

**Authors:** Rebeca Vieira Costa, Karolyny Martins Balbinot, Gabriela Cristina Avertano Rocha da Silveira, Maria Sueli da Silva Kataoka, Silvio Augusto Fernandes de Menezes, Vanessa Morais Freitas, Ana Carolina Uchoa Vasconcelos, Adriana Etges, Victor Angelo Martins Montalli, Fabricio Passador Santos, Sérgio de Melo Alves Júnior, Ruy Gastaldoni Jaeger, João de Jesus Viana Pinheiro

**Affiliations:** 1 Laboratory of Pathological Anatomy and Immunohistochemistry, School of Dentistry, Federal University of Pará, Rua Augusto Corrêa, 01, Belém, Pará 66075-110 Brazil; 2 Cell Culture Laboratory, School of Dentistry, Federal University of Pará, Rua Augusto Corrêa, 01, Belém, Pará 66075-110 Brazil; 3 Department of Periodontics, University Center of Pará, Belém, PA Brazil; 4 Department of Cell and Developmental Biology, Institute of Biomedical Sciences, University of São Paulo, São Paulo, Brazil; 5 Center for the Diagnosis of Diseases of the Mouth, School of Dentistry, Federal University of Pelotas, Rua Gonçalves Chaves, 457/607, Pelotas, Rio Grande do Sul 96015-560 Brazil; 6 Department of Oral Pathology, São Leopoldo Mandic Institute and Research Center, Rua Dr. José Rocha Junqueira, 13-Pte. Preta, Campinas, SP 13045-755 Brazil

**Keywords:** Ameloblastoma, Immunohistochemistry, Cell proliferation, Apoptosis, Pathology, Pathogenesis, Oncogenesis

## Abstract

The aim of this study was to verify whether the expression of cell proliferation and apoptosis markers in different types of unicystic ameloblastoma (UA) is associated with the location of neoplastic cells. Immunohistochemical study with a sample of 32 cases of UA, 11 cases of conventional ameloblastoma (CAM) and ten dental follicles (DF) cases was performed. Cell proliferation was assessed using Ki-67 status, and apoptosis by caspase-3 expression. Mural UA (MUA) showed a higher immunostaining of Ki-67 (*p* < 0.05) and a lower immunostaining of Caspase-3 (*p* < 0.05) compared with luminal and intraluminal subtypes of UA and CAM. The neoplastic cells of the MUA’s cystic capsule showed a higher expression of Ki-67 protein (*p* < 0.0001) and a lower expression of Caspase-3 (*p* < 0.0001) compared with the lumen. DF showed lower Ki-67 and Caspase-3 immunostaining (*p* < 0.05) than neoplasms. The higher immunoexpression of Ki-67 and the lower immunoexpression of Caspase-3 in MUA, in the parenchyma cells within the cystic capsule, suggest an association between the biological behaviour and location of neoplastic cells in a tumour.

## Introduction

Ameloblastoma is the most prevalent benign odontogenic tumour^1^, it has an ectodermal origin and is characterised by the proliferation of the odontogenic epithelium without ectomesenchyme. Furthermore, in line with the guidelines of the World Health Organization (WHO)^2^, although it presents a slow growth pattern, ameloblastoma is considered an aggressive tumour, due to its capacity for local tissue invasion and destruction. Among the variations of this tumour, conventional ameloblastoma (CAM) is the most common, followed by unicystic ameloblastoma (UA)^3^.

During clinical and radiographic examination, UA presents as a cyst; however, on histological examination, a typical ameloblastic epithelium is observed^3^, and the proliferation site in the cystic cavity denominates three other histopathological subtypes. These include the luminal unicystic ameloblastoma (LUA)^4^, where the tumour is confined to the luminal surface of the cyst and the lesion has a fibrous cystic wall, with a lining consisting entirely or partially of the ameloblastic epithelium; intraluminal unicystic ameloblastoma (IUA), which has one or more nodules projecting from the cystic lining towards the lumen; and mural unicystic ameloblastoma (MUA), which demonstrates greater invasiveness by infiltrating the fibrous wall of the cyst and therefore exhibits a more aggressive behaviour compared with the other subtypes^4^. In the 2017 classification^5^, the possibility of changing the re-labelling of the category of the MUA subtype from unicystic ameloblastoma to conventional ameloblastoma was raised, based on the need for aggressive surgical treatment for both type of lesions. In the 2022 classification^2^, MUA was categorised under UAs. Interestingly, CAM and UA mutations of BRAF proto-oncogene mutation V600E (BRAFp. V600E) were found in several cases. However, only CAM showed additional mutations^6^, which potentially explains CAM’s clinical behaviour, but not that of MUA.

There are significant differences in the biological behaviour between these variants, which implies the choice of either conservative or aggressive treatments, such as surgical resection of the lesions with safety margins, as well as the prognosis of the lesions. The variations in their biological behaviour may be attributed to the expression of proteins related to tumour invasion, infiltration and the destruction of healthy tissue. Furthermore, these proteins are found to adhere to and destroy the extracellular matrix^7^. The various mechanisms of the typical biological behaviour of tumours with cystic areas include cell proliferation and apoptosis. It has been reported that there are more apoptosis-inhibiting proteins than apoptosis-promoting proteins in CAM^8^.

The process of cell proliferation impacts tissue homeostasis as well as several biological and pathological events, such as the development of tumours. The identification of cell proliferation markers can be an effective diagnostic method to understand and predict the biological behaviour of several lesions. A marker of cell proliferation that stands out in studies is the Ki-67 protein, whose expression is observed in several phases of the cell cycle and disappears after mitosis^9^.

In contrast to cell proliferation, apoptosis occurs through the activation of members of the family of cysteine aspartic proteases (caspases), such as Caspase-3, which in its active form is considered a marker of apoptosis, due to the effector function in this biological phenomenon^10^. It is believed that the formation of ameloblastomas may be caused by an imbalance between cell proliferation and cell death, but little is known about their aetiopathogenesis of their formation^10^.

In previous studies^11,12^, we hypothesised that neoplastic epithelial cells of CAM that are close to the bone would present a higher rate of proliferation and be stimulated by growth factors released by bone resorption to synthesise and secrete metalloproteinases. The present study aims to evaluate the immunoexpression rates of Ki-67 and Caspase-3 proteins in UA subtypes, CAM and DF, and to verify the differences in the immunostaining of these proteins between the cells located in the lumen and cystic capsule regions of MUA since the neoplastic epithelial cells of MUA are closer to the bone being reabsorbed by the neoplasm. Therefore, there is an urgent need to gain a deeper insight into the mechanisms contributing to the difference in the biological behaviour of each subtype. This is essential to directing appropriate treatment approaches for each UA subtype. In this study, we hypothesised that neoplastic cells from the cystic capsule region in MUA would have higher immunostaining rates for Ki-67 and lower immunostaining rates for Caspase-3 due to their proximity to the bone undergoing resorption.

## Methods

### Study design and ethical approval

The Ethics Committee in Research with Human Beings of the Institute of Health Sciences of the Federal University of Pará – ICS/UFPA has waived the need for informed Consent to Participate due to the retrospective nature of the study and approved this research under protocol number 4,570,860. The Declaration of Helsinki guidelines were followed^13^. Immunohistochemical reactions were performed on 53 human-derived samples, including 32 UA samples, 11 primary CAM samples and ten DF samples.

The samples and clinical data of the patients were collected from the archives of the Centre for the Diagnosis of Mouth Diseases (CDDB) of the Faculty of Dentistry at the Federal University of Pelotas, the São Leopoldo Mandic Research Centre and Institute, and the Laboratory of Pathological Anatomy and Immunohistochemistry of the Faculty of Dentistry at the Federal University of Pará. All samples were diagnosed based on imaging analysis and trans-surgical exams, combined with the histological analysis of the entire lesion to rule out an invasion of tumour epithelium into the cystic capsule in cases of UA.

### Sample

The total sample was divided into five groups, according to the 2022 WHO classification for head and neck tumours: the MUA group with 18 samples of the mural subtype; the LUA group comprising eight samples of the luminal subtype; the IUA group with six samples of the intraluminal subtype; the CAM group consisting of 11 samples microscopically diagnosed as conventional ameloblastoma, which served as a positive control, owing to the established expression of Ki-67 and Caspase-3 proteins in this tumour^14–16^; and the DF group, consisting of ten samples of dental follicles, which are normal dental tissue that does not characterise cystic or neoplastic alterations^2^. Following the same diagnostic pattern, all cases of UA and CAM were diagnosed based on the clinical, radiographic, surgical and histopathological aspects^3^.

For immunohistochemical evaluation, the loaded histological slides were deparaffinised and washed with xylene dehydrating ethanol solution. Subsequently, the samples were immersed in 3% hydrogen peroxide and methanol (1:1) to block endogenous peroxidase activity. Antigen retrieval was performed using citrate buffer (pH 6.0) in a pressure chamber (Pascal pressure chamber; Dako Cytomation, Carpinteria, CA, USA) for 30 s at 125 °C. After treatment with 1% bovine serum albumin (Bovine serum albumin; Sigma-Aldrich, St. Louis, MO, USA) in phosphate-buffered saline solution for 1 h, the sections were incubated for 1 h in a humid chamber at room temperature with the primary antibodies, anti-human Ki-67 (monoclonal antibody rabbit anti-human Ki-67, Clone SP6; Spring Bioscience, Pleasanton, CA, USA), diluted 1:25, incubated overnight, and anti-human caspase-3 (Activated Rabbit Anti Caspase-3 Polyclonal Antibody, CPP32; Diagnostic BioSystem, Pleasanton, CA, USA), diluted at 1:600 for 1 h and incubated separately. Slides were incubated and treated at room temperature with a dextran polymer–based complex (Reveal; Spring Bioscience, Pleasanton, CA, USA), and diaminobenzidine (DAB) was used as a chromogenic agent (Liquid DAB + Substrate; Spring Bioscience, Pleasanton, CA, USA). Finally, the slides were counterstained with haematoxylin (Mayer’s haematoxylin; Sigma-Aldrich, St. Louis, MO, USA) and were mounted with mounting medium (Permount; Fisher Scientific, Fair Lawn, NJ, USA). CAM samples were used as a positive control, and a negative control was performed with the omission of the primary antibody, replaced by non-immune serum.

From each sample, a total of five images were acquired randomly using a microscope (AxioScope; Carl Zeiss, Oberkochen, Germany) equipped with a colour camera (AxioCam HRC; Carl Zeis, Oberkochen, Germany) at 400 × magnification. The DAB-immunomarked nuclei in the tumour parenchyma were counted using a specific plugin of imaging software (Cell Counter; ImageJ, NIMH, Bethesda, MD, USA). The number of cells in the tumour parenchyma was counted to verify the percentage of nuclear immunostaining (labelling index) according to Siqueira et al^12^. In the case of MUA, 10 images of each sample were acquired: 5 images for cells that invaded the cystic capsule and 5 for cells present in the lumen for a comparison between these areas.

### Data analysis

The data obtained were analysed using a statistics software (GraphPadPrism 8 software; GraphPad Software, San Diego, CA, USA) and by the statistics software (jamovi 2.3; The jamovi project, Sydney, NSW, Australia). A non-parametric distribution was evidenced in the Shapiro–Wilk test; thus, the differences between the groups were evaluated by the ANOVA test with Bonferroni correction. The difference between the MUA capsule and lumen areas was estimated using the Mann–Whitney test. A 95% confidence interval was assumed (*p* = 0.05).

### Ethics approval and consent to participate

This study was carried out as per the criteria established by the Ethics Committee in Research with Human Beings of the Institute of Health Sciences of the Federal University of Pará—ICS/UFPA. 

The Ethics Committee in Research with Human Beings of the Institute of Health Sciences of the Federal University of Pará—ICS/UFPA waived the Consent to Participate form and approved this research under protocol number 4,570,860.

## Results

The samples of the different types of ameloblastoma were distributed into groups after collecting clinical and anatomopathological data, which can be seen in Table 1.

**Table 1:** Clinical and demographic characteristics of the ameloblastoma samples: histological subtype, sex, age and anatomical location of NSW the lesion.

Variables	Unicystic ameloblastoma			Conventional ameloblastoma	Dental follicle
	Luminal	Intraluminal	Mural		
Sex (n = 53)					
Female	6	2	9	5	4
Male	2	3	6	6	6
NR*	0	1	3	0	0
Age (n = 53)					
00 − 09	0	2	2	0	0
10 − 19	3	2	2	1	5
20 − 29	4	1	7	2	2
30 − 39	0	0	3	4	1
40 − 49	0	0	0	2	0
50 − 59	1	0	0	2	0
60 − 69	0	0	0	0	0
70 − 79	0	0	0	0	0
80 − 89	0	0	0	0	0
NR*	0	1	4	0	2
Anatomical Location (n = 53)					
Maxilla	0	0	1	0	0
Mandible	8	5	14	11	8
NR*	0	1	3	0	2

NR* = Not Reported.

**Figure 1:**
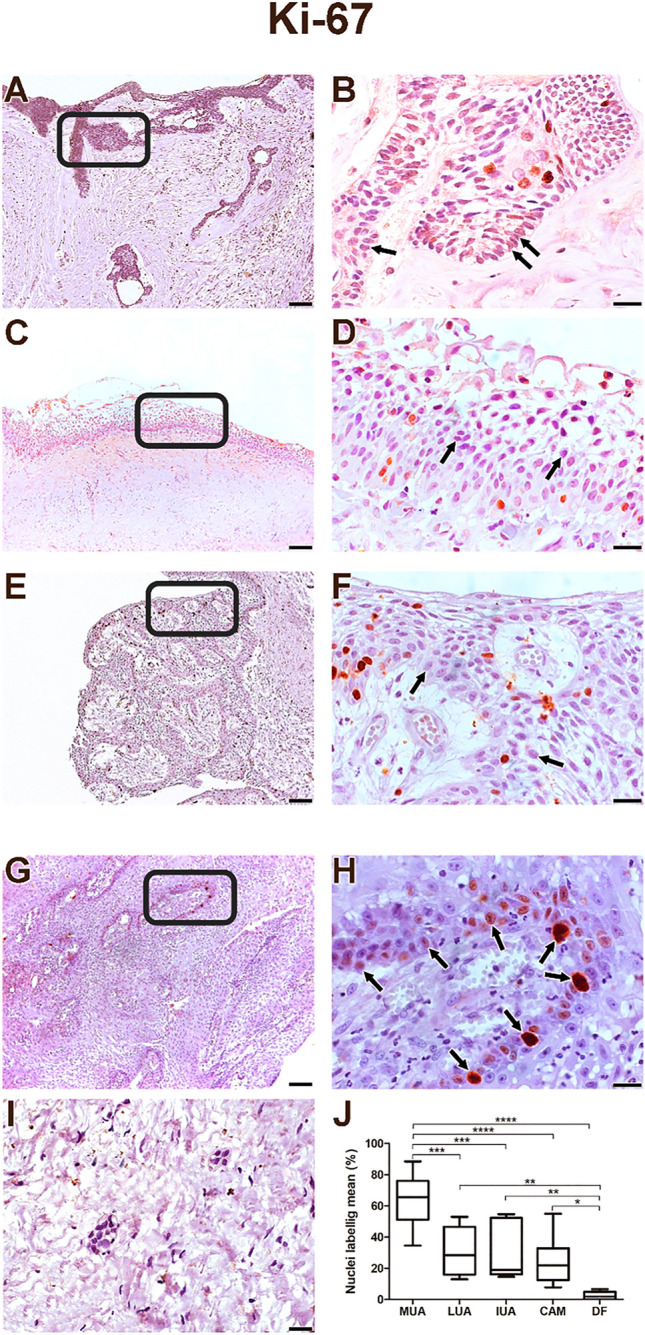
Proliferating marker Ki-67 is higher on MUA (**A**-**B**) in comparison with LUA (**C**-**D**), IUA (**E**–**F**), CAM (**G**-**H**) and DF (**I**) samples. The markings were predominantly nuclear in the neoplastic cell parenchyma. Scales: 20 µm and 100 µm. Comparison of Ki-67 immunoexpression between the samples of MUA, LUA, IUA, CAM and DF, ****p < 0.0001, ***p < 0.001, **p < 0.01, *p < 0.05 (J). In figures B and H the immunostaining is observed mainly in the tall columnar cells on the periphery of the tumor islands (arrow). In figures D and F a weak staining is observed in most neoplastic cells (arrows).

**Figure 2:**
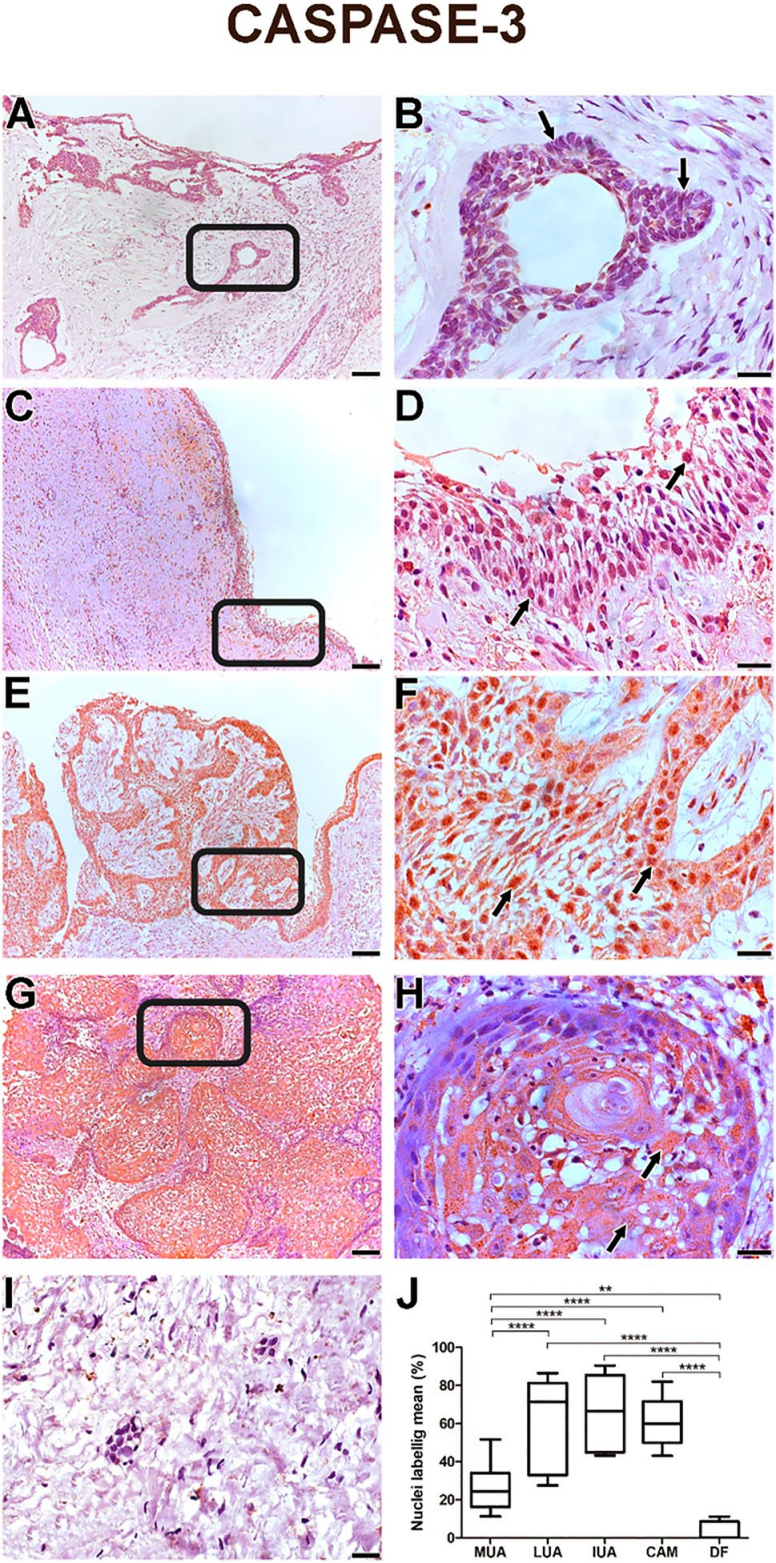
Apoptosis marker Caspase-3 is lower in MUA (**A**, **B**) than in LUA (**C**, **D**), IUA (**E**, **F**), and CAM (**G**, **H**), except for DF (**I**). For LUA (**C**, **D**), IUA (**E**, **F**) and CAM (**G**, **H**), Capase-3 is higher than DF (**I**). The markings were weakly diffused into the cytoplasm in the parenchyma of neoplastic cells. Scales: 20 µm and 100 µm. Comparison of the immunoexpression of Caspase-3 between the samples of MUA, LUA, IUA, CAM and DF, ***p < 0.0001, **p < 0.001, *p < 0.05 (J). In figure B, immunostaining is barely observed in the tall columnar cells at the periphery of the tumor islands (arrow). In figures E and F, strong staining is observed in the many of neoplastic cells (arrows).

All samples from the CAM, MUA, LUA, IUA and DF groups showed an expression of Ki-67 and Caspase-3 proteins in neoplastic parenchyma cells. The immunohistochemical staining of the studied proteins was in the cords and islands of the odontogenic tumour epithelium. Ki-67 labelling was predominantly nuclear (Fig. 1, A–H). Caspase-3 labelling was verified in the cell nucleus and found to be diffused in the cytoplasm of tumour parenchyma cells (Fig. 2, A–H). MUA had a higher Ki-67 immunostaining rate and a lower Caspase-3 rate compared with LUA (Ki-67: *p* < 0.001; Caspase-3: *p* < 0.0001), IUA (Ki-67: *p* < 0.001; Caspase-3: *p* < 0.0001) and CAM (Ki-67: *p* < 0.0001; Caspase-3: *p* < 0.0001). For both proteins, the LUA, IUA and CAM groups showed no statistical difference (Figs. 1J and 2J).

**Figure 3:**
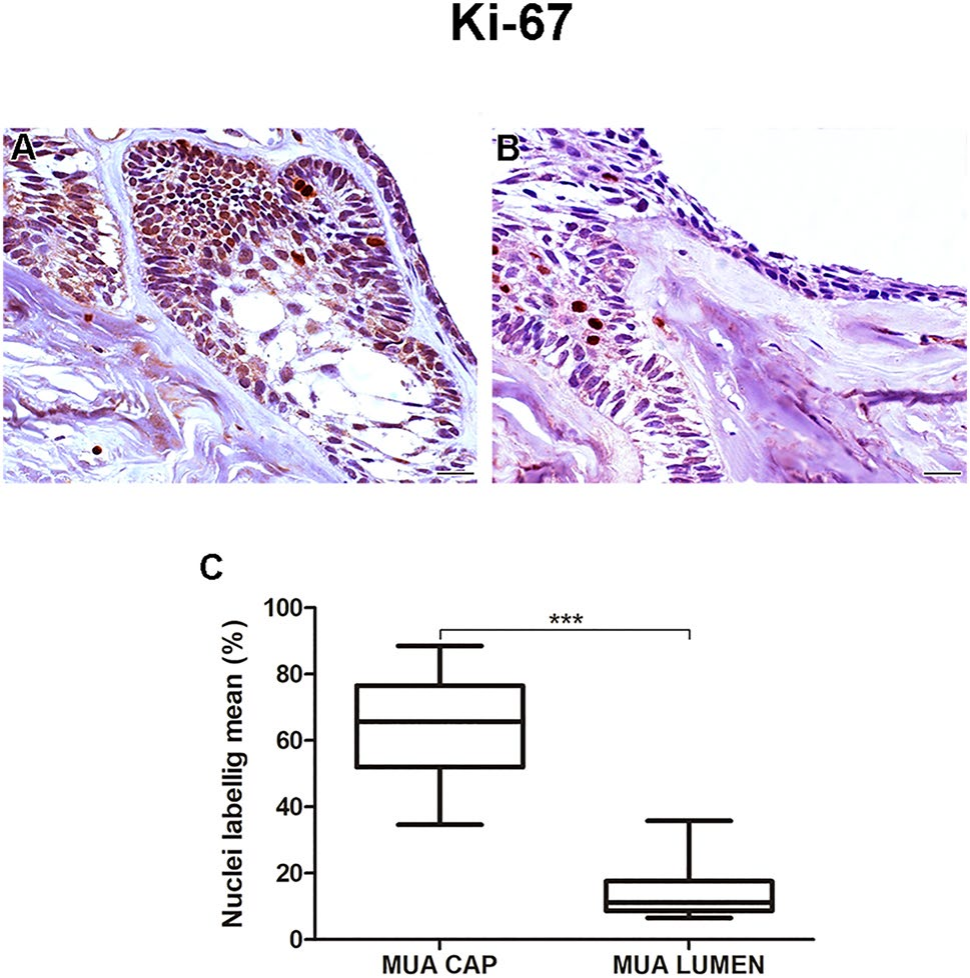
Proliferating marker Ki-67 is higher on (**A**) neoplastic MUA cells in the cystic capsule region compared with (**B**) neoplastic MUA cells in the lumen region. Scale: 50 µm. (**C**) Comparison of Ki-67 immunoexpression between the capsule and lumen areas of MUA, ***p < 0.0001.

**Figure 4:**
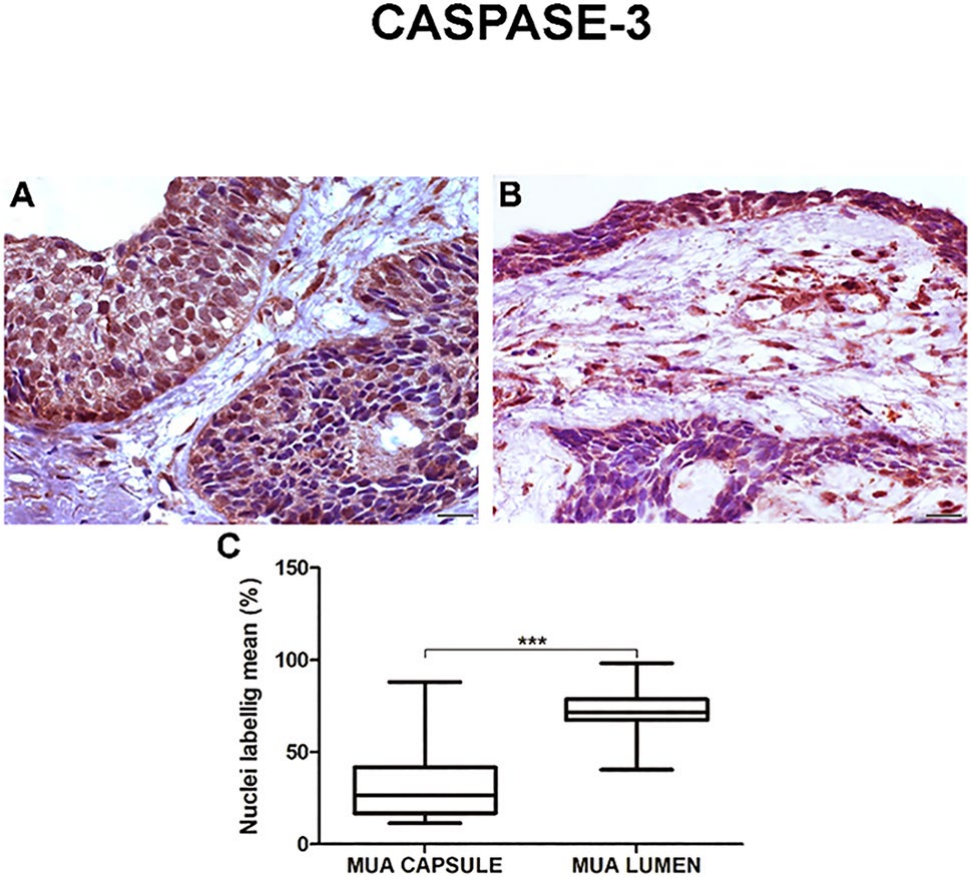
Apoptosis marker Caspase-3 is lower in (**A**) neoplastic MUA cells in the cystic capsule region in comparison with (**B**) neoplastic MUA cells in the lumen region. Scale: 20 µm (**C**). Comparison of Caspase-3 immunoexpression between the capsule and lumen areas of MUA, ***p < 0.0001.

The neoplastic cells of the MUA’s (Figs. 3A, B and 4A, B) cystic capsule showed a higher percentage of Ki-67 protein expression (*p* < 0.001) and a lower percentage of Caspase-3 expression (*p* < 0.001) compared with the lumen (Figs. 3C and 4C). DF (Figs. 1I and 2I) showed lower Ki-67 and Caspase-3 immunostaining rates than MUA (Ki-67: *p* < 0.0001; Caspase-3: *p* = 0.004), LUA (Ki-67: *p* = 0.002; Caspase-3: *p* < 0.0001), IUA (Ki-67: *p* = 0.008; Caspase-3: *p* < 0.0001) and CAM (Ki-67: *p* = 0.011;d Caspase-3: *p* < 0.0001) (Figs. 1J and 2J).

## Discussion

In this study, we hypothesise that MUA has a higher Ki-67 expression (proliferation marker) and lower Caspase-3 expression (apoptosis marker) than unicystic ameloblastoma subtypes, due to the location of neoplastic cells in MUA. On analysis, neoplastic cells from the cystic capsule region showed higher Ki-67 immunoexpression and lower Caspase-3 expression, when compared with neoplastic cells from the cystic lumen region in MUA, LUA and IUA.

The clinical data of the studied UA samples displayed a higher prevalence of the mural subtype, with most patients aged 25 years or less and being female. The most prevalent site was in the mandible region. In the CAM samples, patients were predominantly over 25 years of age, and the predominant location was the mandible.

Research has observed a higher prevalence of UAs in young patients^17,18^, in the third decade of life, and a higher incidence in the posterior region of the mandible, and most of them were classified as the mural variant. In one study^19^, CAM was found to be more frequent in patients who were in their third to seventh decade of life, and the mandible was the most affected location. The clinical data generated the present study corroborate the findings in the literature.

In the immunohistochemical evaluation of the expression of Ki-67 and Caspase-3 proteins, in the neoplastic parenchyma of the UA and CAM variants, the labelling of the proteins was studied in all samples. When evaluating the predominance of nuclear labelling of the Ki-67 protein, the mural subtype was found to show a higher immunoexpression of this protein than the other subtypes and CAM. In contrast, the Caspase-3 protein was less expressed in the parenchyma of MUA, compared with the other subtypes and CAM. Meanwhile, LUA and IUA showed no difference of caspase-3 expression when compared with CAM.

Some studies have shown that the central cells in CAM that resemble the stellate reticulum of the enamel organ tend to undergo apoptosis, showing a greater expression of Caspase-3^20^. The greater expression of Caspase-3 in this region has been related to hypoxia due to less local vascularization^10,21^. On the other hand, tall columnar cells in the periphery demonstrate a higher expression of Ki-67^20^. The tall columnar cells in the periphery, because they are in contact with the extracellular matrix and/or close to the surrounding bone, would likely receive stimuli for proliferation with the release of growth factors by these structures. Additionally, a study demonstrated, using selected samples of CAM, that the neoplastic cells of ameloblastoma, when closer to the surrounding bone, have a higher proliferation rate^11^. Thus, based on the above studies, we can extrapolate that in random samples, epithelial cells of CAM may be stimulated to proliferate when they are close to the bone and to cause apoptosis when they are far from the surrounding bone or in the centre of islands and cords. This probably does not occur with MUA cells that have invaded the capsule, as these are mainly characterised by cells from the basal layer, close to the surrounding bone, resulting in a higher expression of Ki-67 and a lower expression of Caspase-3 than in CAM.

Additionally, the present study found that CAM did not present any statistical difference when compared with LUA and IUA for both studied proteins. This may be because there are possibly different cellular mechanisms associated with other pathways related to the invasiveness of the and perhaps Ki-67 and Caspase-3 have a greater role in the proliferation and progression in unicystic ameoblastomas, not characterising the real capacity of CAM local invasion. For example, it is known that in CAM, the activated Mitogen-activated protein kinases (MAPK) pathway, the BRAFp. V600E and additional mutations activate signalling pathways such as mitogen-activated protein kinase kinase (MEK)/extracellular signal-regulated kinases (ERK)^6^ that potentiate phenomena related to tumour invasiveness such as the secretion of metalloproteinases (MMPs)^11^, cell differentiation and angiogenesis^22^.

Another hypothesis to justify this result is that the histopathological sections analysed were not performed in selected areas and possibly represent more central areas of CAM, and in theory, the release of mitogenic factors would occur in the most peripheral location of the tumour, favouring cell proliferation and limiting defence mechanisms, such as apoptosis, which is seen in more central regions in ameloblastoma^20,21^.

The hypothesis proposed in this study suggest that the cells closest to the bone have a higher expression of Ki-67 and a lower expression of Caspase-3, which could explain the lack of statistical difference between CAM, LUA and IUA.

The Ki-67 immunostaining index in cells is related to proliferation, local invasion, and it constitutes a prognostic factor^16^. In two studies that used immunohistochemistry, the authors reported no difference in the rates of nuclear staining of Ki-67 between UA and CAM^23,24^. However, one of them did not perform a comparative study on Ki-67 immunoexpression between UA subtypes^24^. The other one, when comparing unicystic variants, found a greater expression of proteins in the luminal subtype^23^. The authors justify this finding by suggesting the possibility that in this subtype, there are fewer stellate reticulum–like cells and consequently most of the cells counted corresponded to those from the basal or suprabasal layers, which are more likely to be positive^23^.

Another study analysing the expression of Ki-67 in UA subtypes has shown higher values in the mural and intraluminal variants than in the luminal variant^4^. In the present study, Ki-67 was most expressed in MUA when compared with the LUA, IUA and CAM groups. The higher expression of this protein typically implies a greater potential for cell proliferation, which would explain why MUA has a greater tendency towards local invasiveness compared with other UA variants. Furthermore, in our research, it was observed that MUA has a higher Ki-67 labelling index than CAM. This may be explained by the fact that the neoplastic epithelial cells in UA that invade the capsule are closer to the surrounding bone, while those in CAM are both close and far from the bone, when they are located in more central areas. However, these cells are equidistant from the surrounding bone matrix, so they would not receive as much stimulus resulting from bone resorption. Additionally, some studies have shown that in tumour microenvironments with hypoxia, there is an increase in apoptosis and the formation of cystic areas in the central portions of CAM^10,21,25^.

**Figure 5:**
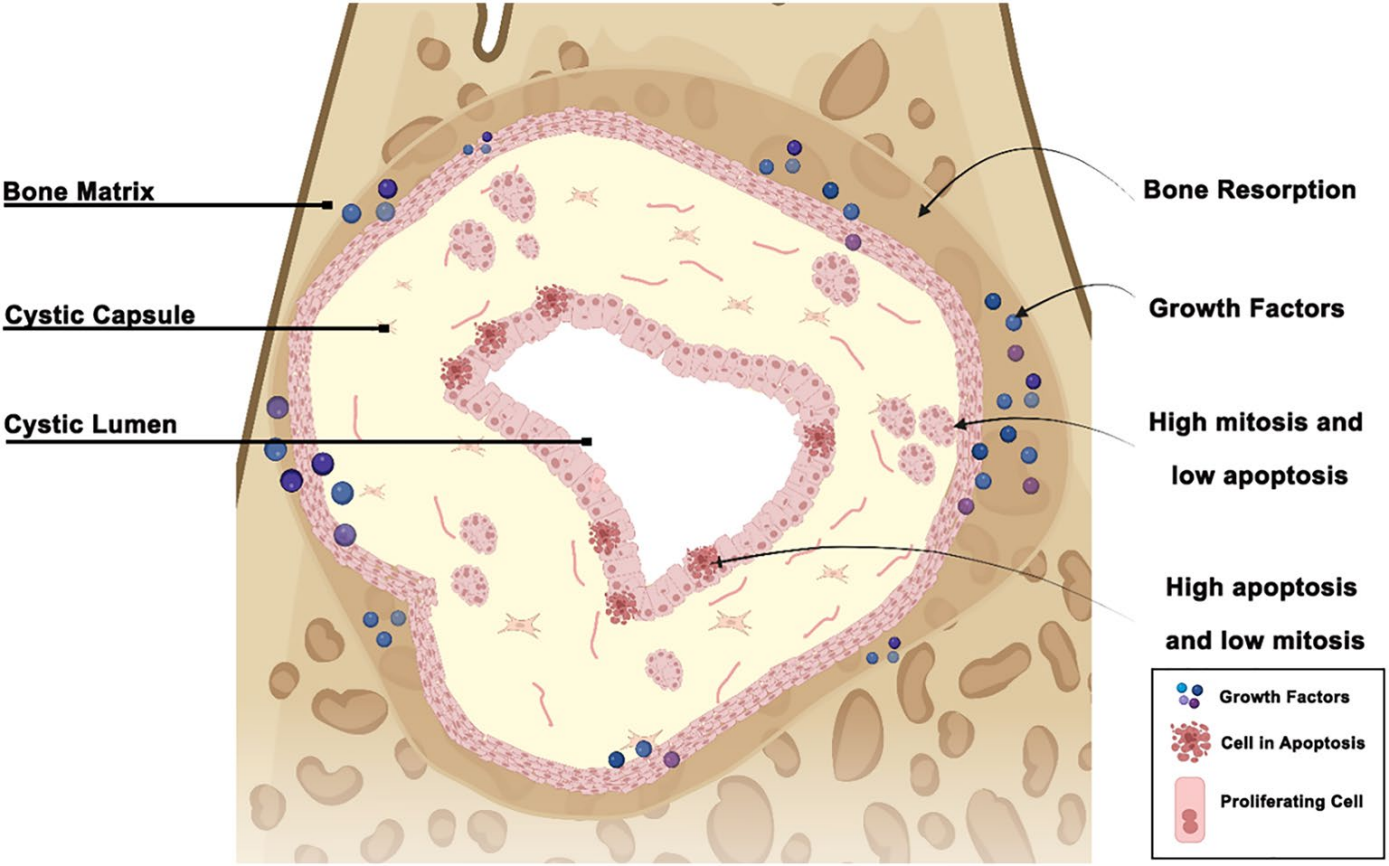
Illustration summarising our hypothesis about the difference between the location of neoplastic cells in mural unicystic ameloblastoma. Neoplastic AUM cells are present both in the lumen and the cystic capsule, with those in the latter being closer to the area of bone degradation than those in the lumen. Created with BioRender.com.

The cells with the most expressive markings in MUA was the neoplastic cells of the cystic capsule. Since the parenchymal cells of MUA are closer to the bone surrounding the cyst, these results can be justified by studies that state that both neoplastic and stromal cells secrete metalloproteinases, which degrade the bone matrix and cause an additional release of growth factors. These mitogens, in turn, are released randomly and are likely to increase the rate of cell proliferation of ameloblastoma parenchyma cells^11,12^ (Fig. 5). Incidentally, the DF samples showed lower Ki-67 and Caspase-3 immunostaining rates compared with the other groups. It is thus understood that the fact that DF epithelial nests do not show a neoplastic growth pattern has led to this result.

In this study, the immunoexpression rate of the pro-apoptotic protein, Caspase-3, in different types of ameloblastoma was evaluated. Statistical difference was observed when comparing the expression of this protein in the mural subtype, with the lowest rates, that in the other UA subtypes. The lower expression of Caspase-3 in MUA may result in a longer survival of tumour parenchyma cells, which possibly influences their biological behaviour. From what could be observed, so far, this is an unprecedented finding in the literature. A study on tumour necrosis factor–related apoptosis-inducing ligand (TRAIL) reported that ameloblastoma has an anti-apoptotic proliferation site in the periphery and a pro-apoptotic differentiation site in the centre of the parenchyma tumour^8^.

These results can possibly explain the difference in Caspase-3 expression between MUA and CAM found in the present study. The lower expression in the parenchyma cells present in the cystic capsule of MUA probably contradicts the higher expression in the centre of CAM. In addition, tumour cells typically demonstrate a high demand for oxygen during proliferation, and the restriction of access to oxygen in the central area of the tumour leads to the creation of hypoxic microenvironments^10^. This process occurs in most solid tumours and can result in an increase in aggressiveness at the front of tumour invasion (tumour periphery)^26^ or lead to tumour death by apoptosis in the centre of the tumour^21^. This validates the result of quantification in different regions of the MUA, in which the highest rates of immunostaining were found for the apoptosis-promoting protein in the cystic lumen region (tumour centre).

Some studies have also concluded that there are more apoptosis-inhibiting proteins than apoptosis-activating proteins, suggesting that conventional ameloblastoma parenchyma cells may have a high survival activity and thus a high recurrence rate^8,21,27,28^. This can be attributed to a greater proliferation of CAM at the bone–neoplasm interface than at the centre of this tumour^21,25^.

By silencing an anti-apoptotic protein (Bcl-2) in an assay with ameloblastoma cells, the authors of this study observed an inhibition of tumour formation in vivo and suggested that studying apoptosis modulators may favour the prediction of recurrences and the development of effective chemotherapeutic agents for treating these lesions^27^. The behaviour of ameloblastoma is closely related to the potential of tumour cells to inhibit apoptosis, and it is also associated with the ability to initiate a proliferative phase of lesions^28^.

Still, in this context, the literature has pointed out that growth factors such as fibroblast growth factor (FGF), keratinocytes growth factor (KGF), epidermal growth factor (EGF) and transforming growth factor beta (TGF-β), among others, participate and are released during bone remodelling/resorption^29^. A major proportion of the literature argues that these molecules may have an anti-apoptotic effect^30–33^. Thus, the growth factors would also possibly act by inhibiting apoptosis on the invasion front of MUA parenchyma cells. In addition to growth factors acting against apoptosis, mutations in the smoothened frizzled class receptor (SMO) and BRAF genes participate in the FGF pathway, which is associated with this phenomenon^34^. However, UA does not have additional mutations to BRAFp.V600E^6^. Therefore, only the BRAFp.V600E mutation, in cases of UA, would not justify the clinical behaviour of MUA.

The low expression of Caspase-3 in DF, which is a non-neoplastic tissue, as well as in UA, indicates anti-apoptotic events overlap apoptotic ones in this tumour. The expression in LUA, IUA and CAM could be high because they are not largely influenced by growth factors and genetic mutations, related to the inhibition of apoptosis.

The mentioned studies indicate the probable role of anti-apoptotic modulators in the local invasiveness of ameloblastoma, although no study has verified the expression of Caspase-3 in UA and its subtypes.

Since the method used for the development of this immunohistochemistry study is limited, the results must be confirmed against those of studies that investigate the biological mechanisms involved with the findings. In addition, studies with more expressive numbers of UA should be carried out to confirm the results.

In 2022, there was an update in the classification of head and neck tumours, based on the publication made by the WHO^2^. As per the studies carried out by the authors behind the update, ameloblastoma subtypes should be treated conservatively when they are intraluminal and luminal, while the mural subtype should be treated with the same approach used for CAM, as it is associated with recurrences when treated by conservative methods.

In summary, we can extrapolate that the lower Caspase-3 labelling and the higher Ki-67 expression observed in the cystic MUA capsule could justify the more aggressive biological behaviour of this histological subtype.

## Conclusions

The neoplastic cells present in the cystic capsule in the MUAs presented higher expression of the cell proliferation marker and lower expression of the apoptosis marker when compared with neoplastic cells located far from the bone region undergoing resorption. Therefore, bone resorption may be influencing more invasive biological mechanism of MUA compared with other UA subtypes.

## Data Availability

The datasets used and analysed during the current study are available from the corresponding author on reasonable request.
